# Population dynamics of enteric *Salmonella* in response to antimicrobial use in beef feedlot cattle

**DOI:** 10.1038/s41598-017-14751-9

**Published:** 2017-10-30

**Authors:** Naomi Ohta, Keri N. Norman, Bo Norby, Sara D. Lawhon, Javier Vinasco, Henk den Bakker, Guy H. Loneragan, H. Morgan Scott

**Affiliations:** 1 Department of Veterinary Pathobiology, College of Veterinary Medicine and Biomedical Sciences, Texas A&M University, College Station, TX 77843 USA; 2 Department of Veterinary Integrative Biosciences, College of Veterinary Medicine and Biomedical Sciences, Texas A&M University, College Station, TX 77843 USA; 3 Department of Large Animal Clinical Sciences, College of Veterinary Medicine, Michigan State University, East Lansing, MI 48824 USA; 4 Center for Food Safety, College of Agricultural & Environmental Sciences, University of Georgia, Griffin, GA 30223 USA; 5 Department of Animal and Food Sciences, College of Agriculture and Natural Resources, Texas Tech University, Lubbock, TX 79409 USA

## Abstract

A randomized controlled longitudinal field trial was undertaken to assess the effects of injectable ceftiofur crystalline-free acid (CCFA) versus in-feed chlortetracycline on the temporal dynamics of *Salmonella enterica* spp. *enterica* in feedlot cattle. Two replicates of 8 pens (total 176 steers) received one of 4 different regimens. All, or one, out of 11 steers were treated with CCFA on day 0 in 8 pens, with half of the pens later receiving three 5-day regimens of chlortetracycline from day 4 to day 20. *Salmonella* was isolated from faecal samples and antimicrobial susceptibility was analysed via microbroth dilution. Serotype was determined by whole-genome sequencing. On day 0, mean *Salmonella* prevalence was 75.0% and the vast majority of isolates were pansusceptible. Both antimicrobials reduced overall prevalence of *Salmonella*; however, these treatments increased the proportion of multi-drug resistant (MDR) *Salmonella* from day 4 through day 26, which was the last day of faecal collection. Only six *Salmonella* serotypes were detected. *Salmonella* serotype Reading isolates were extensively MDR, suggesting a strong association between serotype and resistance. Our study demonstrates that the selection pressures of a 3^rd^ generation cephalosporin and chlortetracycline during the feeding period contribute to dynamic population shifts between antimicrobial susceptible and resistant *Salmonella*.

## Introduction

Foodborne salmonellosis is estimated to cause more than 1.2 million illnesses annually in the United States, requiring 23,000 hospital admissions and resulting in 450 deaths^1^. Salmonellosis is usually self-limiting, and even severely affected patients generally recover in 5 to 7 days if given rehydration fluids. Antimicrobial treatment options for adults include ceftriaxone, a medically important third-generation cephalosporin, and fluoroquinolones. There are potential side effects of fluoroquinolones for paediatric patients, and hence the first choice of treatment is ceftriaxone^2–4^. Because fewer treatment options are available for antimicrobial-resistant *Salmonella*, infections with these strains are potentially more life threatening^5^. Use of antimicrobials can cause unintentional selection pressure for antimicrobial resistance in the gut microbiota of animals, and therefore can potentially lead to more severe cases of salmonellosis^6–8^.

The National Antimicrobial Resistance Monitoring System (NARMS) has previously reported an increase in ceftriaxone resistant *Salmonella* carrying *bla*_CMY-2_, a class C plasmid and chromosome encoded *amp*C gene, in human cases of salmonellosis^9–11^. The *bla*_CMY-2_ gene also confers resistance to ceftiofur, a third-generation cephalosporin approved for veterinary use, as well as ampicillin, amoxicillin-clavulanic acid, cephalothin, and cefoxitin^9,10^. Observed increases in ceftriaxone resistant *Salmonella* in humans may be due, at least in part, to the increased use of third-generation cephalosporins in food animals^12,13^. This is considered a high public health risk since ceftriaxone and ceftiofur belong to the same class of 3^rd^ generation cephalosporins, which the World Health Organization (WHO) has classified as critically important for human medicine. A variety of risk management strategies have been employed to help maintain antimicrobial efficacy for human medicine and to reduce the spread of antimicrobial-resistant bacteria derived from food animals^14^. In 2008, the U.S. Food and Drug Administration (FDA) announced a plan to prohibit the extra-label use of all cephalosporins in food animals (with no exceptions); later, this was revoked due to concerns about overly broad restrictions and the potential for unintended negative consequences^15^. Following re-examination by the FDA, extra-label use of cephapirin, some extra-label uses for indications involving the same route of administration, dose, and duration, and the use in minor food-producing species were excluded from the 2012 prohibition on extra-label use of cephalosporins in food-producing animals^15^. The FDA also promoted judicious use of antimicrobials of importance to human medicine, by working to remove growth promotion labels as of January 1, 2017^16,17^. The effects of such strategies on reducing human infections with resistant bacteria have yet to be determined^18–20^. In the current study, we investigated treatment strategies involving a 3^rd^ generation cephalosporin and chlortetracycline in fed beef cattle and their effects on selecting for *Salmonella enterica*.

In the recent past, several studies have been conducted to investigate the selection of resistant *E*. *coli* and *Salmonella* with the use of ceftiofur in cattle, both in experimental and observational settings^21–27^. In one study, ceftiofur administration in beef cattle transiently increased ceftiofur-resistant *E*. *coli*; however, the bacterial population returned to the before-treatment level after 2 weeks^22^. Daniels and others showed that ceftiofur use in a dairy herd was not associated with the occurrence of ceftiofur-resistant *Salmonella* and *E*. *coli* ^24^. Singer and others reported that the therapeutic use of ceftiofur in dairy cattle opened the “window” to detect resistant *E*. *coli*, but it was not concluded that such use resulted in the emergence or amplification of resistant *E*. *coli* ^23^. Another dairy farm study reported that the ceftiofur use and *E*. *coli* with reduced susceptibility to ceftriaxone are associated at the herd level, but not at the individual cow level^21^. A 10-month long study by Schmidt *et al*. showed that the ceftiofur use in feedlot cattle did not increase the extended-spectrum cephalosporin resistant *E*. *coli* ^26^. These studies suggest that the therapeutic use of ceftiofur can transiently increase the detection of cephalosporin resistant *E*. *coli*; however, the bacterial population returns to susceptibility after a suitable washout period. Since *E*. *coli* and *Salmonella* belong to the same Enterobacteriaceae family, *Salmonella* seem likely to exhibit a similar response to ceftiofur as *E*. *coli*; however, it is not yet known since studies involving *Salmonella* require consistent presence. An observational study investigating varying levels of ceftiofur use and their association with resistant *Salmonella* isolated on swine farms showed that the barns with rare and common ceftiofur use had 4.1% and 6.0% recovery of *Salmonella* carrying the *bla*_CMY_ gene, respectively, while only 0.15% recovery occurred in the barns with moderate uses of ceftiofur^28^. The results suggested that other factors, such as farm management or environmental factors may be more important than ceftiofur use.

Chlortetracycline (CTC) is a common feed additive used to treat and control bacterial pneumonia (bovine respiratory disease complex) in feedlot cattle; this is in addition to vaccination used to prevent respiratory disease and other injectable antimicrobials used for disease control purposes^29^. In previous work reported by Platt *et al*. from our group, CTC treatment paradoxically reduced the prevalence of ceftiofur resistant *E*. *coli* ^30^. However, contradictory results were found in a subsequent study by Kanwar *et al*. in which CTC treatment increased ceftiofur resistance, most likely due to co-selection^27^. In a longer term study, the effects of prophylactic use of CTC on antimicrobial-resistant *E*. *coli* in beef cattle were studied for 117 days by Agga *et al*.; their findings showed an increased level of tetracycline resistant *E*. *coli* on Day 5 post-treatment, but not on Days 27, 75, and 117^31^. Additionally, prevalence of cephalosporin-resistant *E*. *coli* remained same among CTC and control groups throughout the study period^31^. We have further investigated the antimicrobial resistance profiles of the *Salmonella* population dynamics in response to both ceftiofur and chlortetracycline administration in the very same cattle studied by Kanwar *et al*.^27^. Such a randomized controlled study has not previously been reported concerning antimicrobial resistant *Salmonella enterica* in feedlot cattle.

The current study is focused on the effects of ceftiofur crystalline-free acid (CCFA) and CTC on the prevalence of *Salmonella* in feedlot cattle, and on changes in the profile of antimicrobial susceptibilities of *Salmonella* resulting from the selective pressures of CTC and CCFA. Furthermore, we investigated and report on the temporal dynamics of the *Salmonella* population in response to the use of antimicrobials.

## Methods

### Experimental design

**Figure 1:**
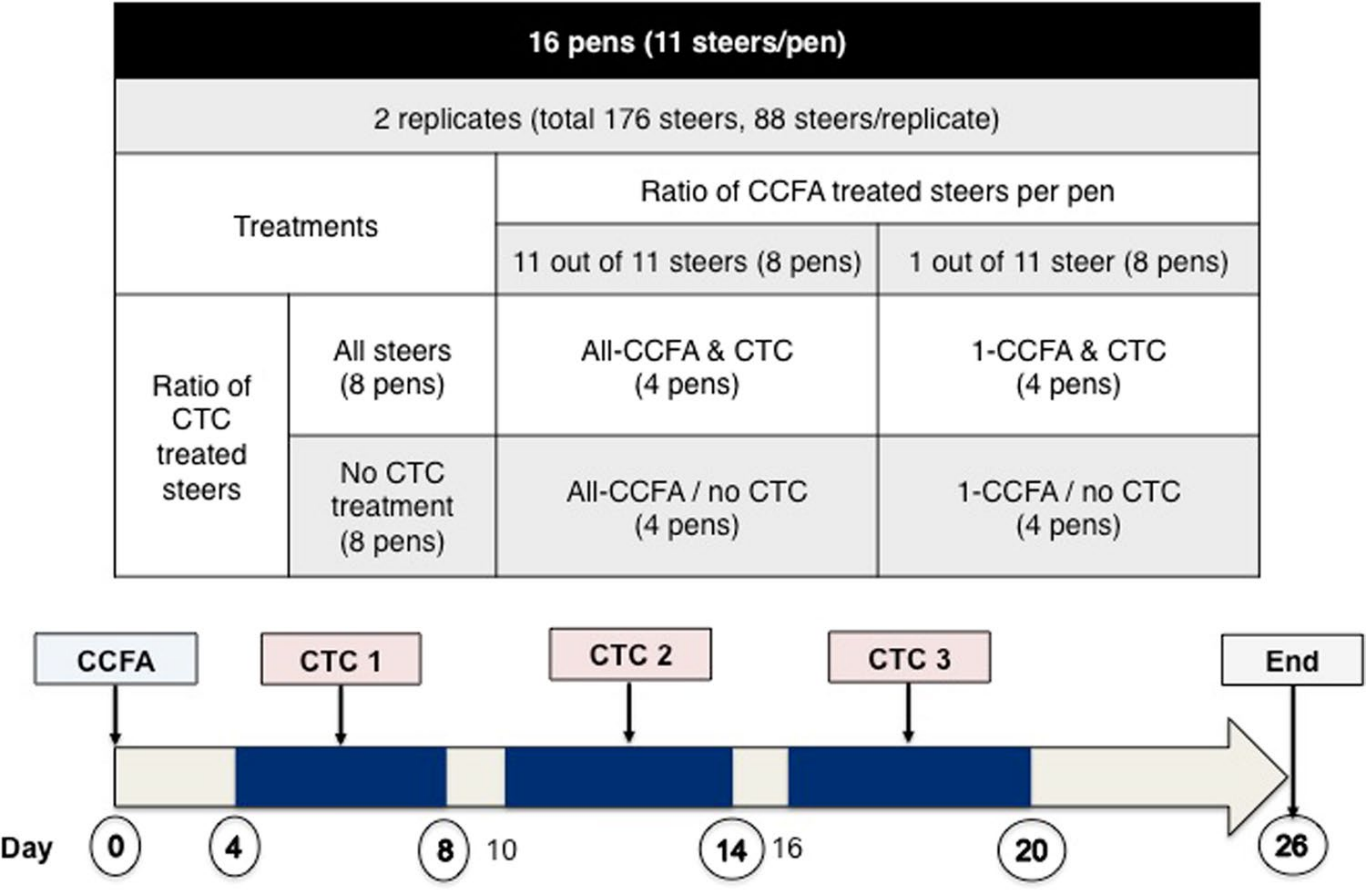
Study design. Four pens were allocated to each treatment over two replicates. Samples were tested from the circled days on the arrow (0, 4, 8, 14, 20, and 26). Un-circled numbers represent the days when ‘pulsed’ CTC was added back into the feed bunks.

A randomized controlled longitudinal field trial was conducted in two sequential 26-day replicates in an experimental feedlot at West Texas A&M University in Canyon, Texas, USA. The first replicate began in early August and a second replicate began in the middle of September. The cattle were owned by a third party, and been purchased from a single operation in the far western United States. They were shipped directly to the experimental feed yard one month before the trial started. It is unknown if the cattle had previously been assembled at the source. They were yearling steers that were predominantly of the Angus breed and were fed diets typical of regional feedlots; that is, a flaked-corned based diet with added roughage, protein, vitamins and minerals. If any cattle became sick and required antimicrobial treatments, they were excluded from the study. In each replicate, 88 steers were assigned into 8 pens (n = 11 cattle) to distribute the body weights among the pens evenly in a two-by-two factorial design with four treatment regimens (Fig. 1), as described in a previous paper by our group^27^. Across both replicates, in 8 pens all 11 steers received 6.6 mg/kg of CCFA (Excede^®^, Zoetis Animal Health, Florham Park, NJ) treatment subcutaneously at the base of the ear (“All-CCFA & CTC” and “All-CCFA/no CTC” in Fig. 1; group metaphylaxis model), and in the remaining 8 pens, a single steer treated with CCFA on day 0 was co-housed (mixed) with 10 non-treated steers. Repeated within each of the two replicates, four of the pens receiving CCFA treatment group later received three 5-day pulses of 22 mg/kg CTC (Aureomycin®, chlortetracycline complex equivalent to 220.5 g/kg of chlortetracycline, Alpharma, Bridgewater, NJ). The CTC was top-dressed in feed with a one-day break between each 5-day pulse. The CTC feeding occurred during the period from day 4 until day 20 (“All-CCFA & CTC”, “1-CCFA & CTC”). The remaining 8 pens in each replicate did not receive CTC (“All-CCFA/no CTC” and “1-CCFA/no CTC”). Faecal samples were collected every other day *per rectum* as described previously^27^. These samples were mixed with glycerol at a 1:1 ratio and preserved at −80 °C. The animal experiments were approved by the Amarillo-Area Cooperative Research, Education, and Extension Triangle Animal Care and Use Committee (Protocol No. 2008-07), and by the Clinical Research Review Committee at Texas A&M University (CRRC # 09–35). All experiments were performed in accordance with institutional and United States Department of Agriculture (USDA) guidelines and regulations governing the oversight and conduct of experiments involving food producing animals in the U.S. Institutional biosafety committee approval # IBC 2014-043 permitted the microbiological laboratory experiments involving *Salmonella enterica* serotypes.

### *Salmonella* isolation from faecal samples

A total of 1,040 faecal samples obtained on days 0, 4, 8, 14, 20, and 26 were tested for the recovery of *Salmonella*. The maximum effect of CCFA treatment for *E*. *coli* isolates being multidrug resistant was seen on day 4 in the previous study^27^, and we expected to observe a similar trend for *Salmonella*. Days 8, 14, and 20 were chosen because these were the last days of each of the 5-day CTC treatment pulses, and we predicted they would reflect the maximum effect on reducing *Salmonella* prevalence. The study was completed on day 26.

We isolated *Salmonella* by following a modified enrichment process as described previously^32^. Samples were thawed on ice and mixed thoroughly with a transfer pipette. In total, 500 mg of faeces were pre-enriched in 5 ml of tryptic soy broth (TSB) (Difco, Becton Dickinson, Franklin Lakes, NJ) for 2 hours at room temperature and then incubated 6 hours at 37 °C and then kept at 4 °C for 14 hours. A 1 ml aliquot of the enriched faeces in TSB was transferred into 9 ml of tetrathionate broth (Difco, Becton Dickinson) and incubated at 37 °C for 18 hours. After incubation, 100 μl of the tetrathionate broth culture was transferred into 10 ml of Rappaport-Vassiliadis R10 (RV) broth (Difco, Becton Dickinson) and incubated at 42 °C overnight. The next day, 50 μl of RV broth was spiral-plated onto Brilliant Green agar (BGA) (Difco, Becton Dickinson) using an Eddy Jet 2 spiral plater (Neutec Group Inc., Farmingdale, NY). Presumptive *Salmonella* isolates were plated on tryptic soy agar (TSA) with 5% sheep blood agar (Remel^TM^, Lenexa, KS) for isolation. Isolates were verified with *Salmonella* O-antiserum Poly A-I & Vi Factors 1–16, 19, 22–25, 34, Vi (Becton, Dickinson and Company, Franklin Lakes, NJ). Confirmed *Salmonella* isolates (n = 566) were preserved in cryobeads at −80 °C for further characterization.

### Antimicrobial susceptibility testing

Minimum inhibitory concentrations (MIC) to 14 antimicrobials (9 antimicrobial classes) of *Salmonella* isolates were determined by the broth microdilution method using the Sensititre^®^ system (TREK, Thermo Scientific Microbiology, Oakwood Village, OH). Tested antimicrobials were ampicillin (AMP), amoxicillin/clavulanic acid (AUG2), azithromycin (AZI), cefoxitin (FOX), ceftiofur (XNL), ceftriaxone (AXO), chloramphenicol (CHL), ciprofloxacin (CIP), gentamicin (GEN), nalidixic acid (NAL), streptomycin (STR), sulfisoxazole (FIS), tetracycline (TET), and trimethoprim/sulfamethoxazole (SXT). Briefly, isolates were plated onto TSA with 5% sheep blood agar and incubated at 37 °C for 18 hours. Then, 1 or 2 colonies were suspended in 4 ml of sterilized water adjusted to 0.5 McFarland standard and 50 µl of the culture suspension was transferred into 11 ml of Mueller-Hinton broth; thereafter, 50 ul of suspension was inoculated onto Gram Negative National Antimicrobial Resistance Monitoring System (NARMS) CMV3AGNF plates using the Sensititre^®^ automated inoculation delivery system (TREK). Plates were incubated at 37 °C for 18 hours. *Escherichia coli* ATCC 25922, *Escherichia coli* ATCC 35218, *Pseudomonas aeruginosa* ATCC 27853, *Staphylococcus aureus* ATCC 29213, and *Enterococcus faecalis* ATCC 29212 (American Type Culture Collection, Manassas, VA) were used as quality control strains for susceptibility testing. Plates were read on a Sensititre OptiRead™ (TREK, Thermo Scientific Microbiology). The results were interpreted as susceptible, intermediate, or resistant according to Clinical and Laboratory Standards Institute (CLSI) guidelines using SWIN software (TREK, Thermo Scientific Microbiology)^33^. When breakpoints were undetermined, we followed the breakpoints established by NARMS for *Salmonella*. Intermediate isolates were reclassified as being susceptible for further statistical analysis. Isolates resistant to three or more classes of antimicrobials were considered multidrug resistant (MDR) as defined by NARMS.

### *Salmonella* DNA extraction for whole-genome sequencing

*Salmonella* DNA was isolated in a QIAcube HT robot using the QIAamp 96 DNA QIAcube HT Kit (Qiagen, Valencia, CA). A single *Salmonella* colony was suspended into 5 ml of TSB and incubated overnight at 37 °C. From the suspension culture, 1 ml was transferred into a 1.2 ml micro-collection tube and centrifuged at 4,000 rpm for 15 minutes at room temperature. After the supernatant was removed, the pellet was re-suspended in ATL buffer (Qiagen) mixed with reagent DX (Qiagen). One tube of small pathogen lysis beads (Qiagen) was mixed with the suspension, and disrupted with the Qiagen TissueLyser system (Qiagen) at 25 Hz, for 5 minutes. The tubes were briefly centrifuged and 40 μl of Proteinase K was added to each tube. The tubes were incubated at 56 °C for 1 hour at 900 rpm in a ThermoMixer (Eppendorf, Hauppauge, NY) followed by a heat shock for 10 minutes at 95 °C. The suspension was cooled to room temperature and 4 μl of RNAse A was added. The prepared samples were set in the QIAcube HT for DNA extraction using a modified protocol provided by Qiagen. The quality of the DNA was determined by the 260/280 ratios on the FLUOstar Omega Microplate Reader (BMG LABTECH, Cary, NC). The DNA quantity was measured with a Quant-iT™ Pico Green^®^ ds DNA Assay kit (Thermo Fisher Scientific) and the DNA was stored at −20 °C until future use.

### Whole-genome sequencing for serotyping and data analysis

To determine the serotypes of the *Salmonella* isolates, we conducted whole-genome sequencing (WGS) using the Illumina MiSeq platform (Illumina, San Diego, CA, USA) on 566 isolates (as described above). Libraries for 32 *Salmonella* DNA samples were multiplexed and pooled per sequence run using the Illumina Nextera XT kit and were run with the MiSeq Reagent Kit v3 with paired-end 2 × 300 bp reads (Illumina). Two *Salmonella* isolates were randomly chosen from each serotype determined by WGS and were sent to the National Veterinary Services Laboratories (NVSL), United States Department of Agriculture (USDA), for traditional serotyping to confirm the sequencing results.

Raw fastq files obtained from the forward and reverse reads were uploaded to the web-based database SeqSero to determine the serotypes from WGS data (http://www.denglab.info/SeqSero)^34^. Multi-Locus Sequence Type (MLST) of each of the *Salmonella* isolates was determined by the combination of 7 gene alleles (*aro*C, *dna*N, *hem*D, *his*D, *pur*E, *suc*A and *thr*A) using the PubMLST database by the SRST2 pipeline in the Illumina® BaseSpace® Sequence Hub^35,36^.

## Statistical Analysis

### Descriptive Statistics

Proportion of *Salmonella* positive (binary outcome) faecal samples and the proportion of isolates resistant to greater than or equal to 3 classes of antimicrobials, or else pansusceptible by sampling day and by treatment group were cross-tabulated in Stata^®^ version 12.1 (StataCorp LLC, College Station, TX). Crude associations between the serotypes, sampling days, and treatment groups were initially tested via the Likelihood-ratio based Chi-Square test or the Fisher’s exact test for rare combinations. Graphics for descriptive statistics were created on Tableau Desktop Professional Edition 10.3.2.

### Multivariable analyses

Prevalence of *Salmonella* (binary) was modelled using a multilevel mixed-effects logistic regression model (-melogit- in Stata^®^ ver. 15.1) considering replicate (2 replicates), pen (16 pens) and animal identifier (176 animals) as potential clustering variables. Following initial assessment of these three potential sources of overdispersion, all three variables were included as significant random effects in the final statistical model. A 3-way full factorial statistical model incorporating fixed effects for 1-CCFA (mixing) and CTC antimicrobial regimens and sampling day was built.

Final model for the prevalence of MDR *Salmonella* (binary) was similarly modelled using multilevel mixed-effects logistic regression (-melogit- in Stata^®^ ver. 15.1). Replicate, pen, and animal identifier were considered as potential clustering variables. Following the initial assessment, replicate was instead included as a fixed effect in the final model, while repeated observations within animal identifier fell out of significance in the presence of pen effects. In addition to replicate, a full factorial statistical model incorporating the fixed effects for each of the CCFA and CTC antimicrobial regimens, sampling day, and 2-way interactions between day and antimicrobial regimens was built.

Marginal mean estimates from the final models were produced and graphical representations of the temporal dynamics were plotted.

### Data Availability

The datasets generated during and/or analysed during the current study are not publicly available due to on-going sequencing analyses at the time of publication; however, they will become available from the corresponding author upon request.

## Results

### Descriptive statistics of *Salmonella* among faecal samples of feedlot cattle

**Figure 2:**
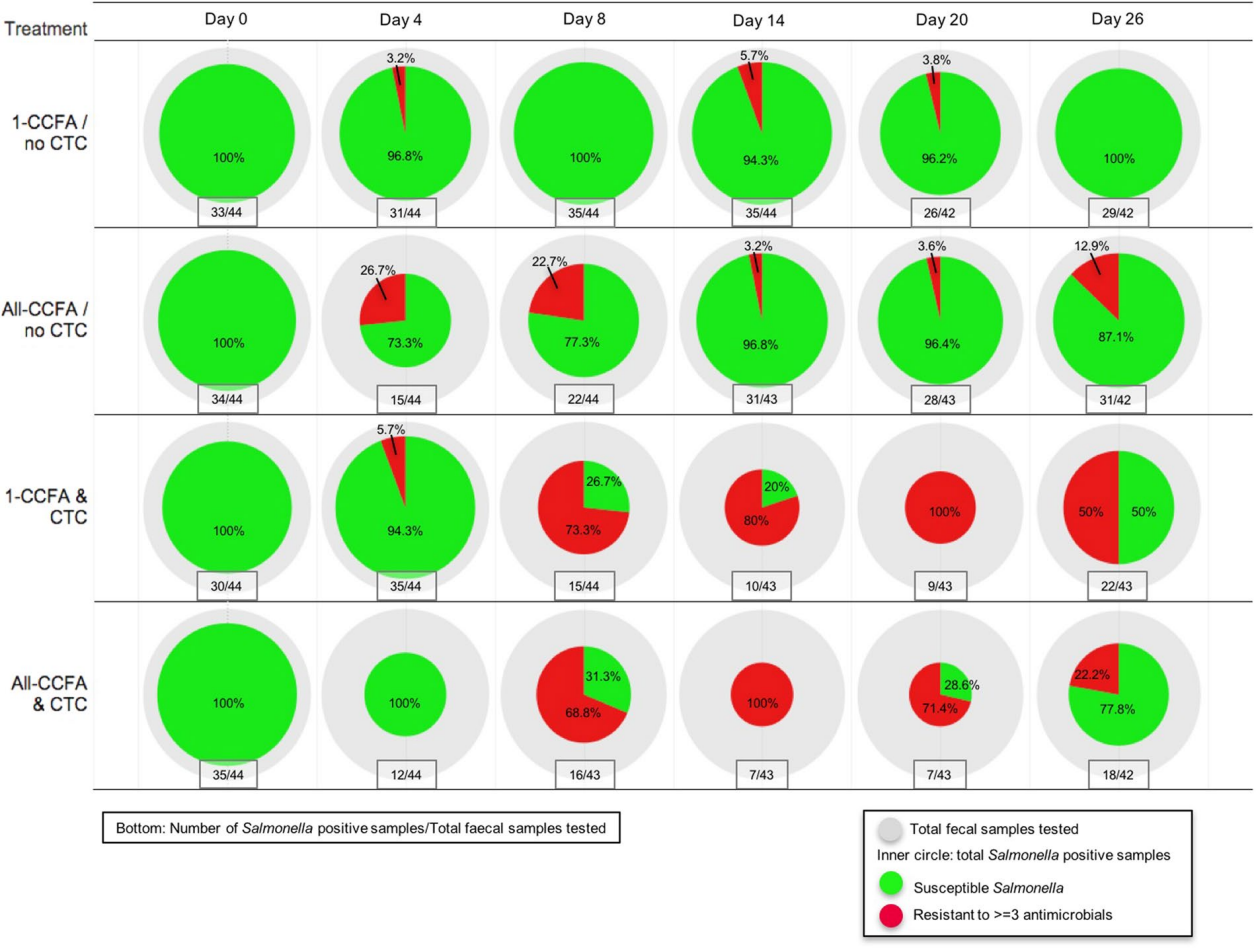
Total number of isolates and proportion of MDR isolates per day and treatment. The outer circle (grey) reflects the number of faecal samples tested per pen each sampling day (n = 42 to 44). The size of the inner circle corresponds to the number of *Salmonella* isolates obtained each sampling day. The green piece of the inner circle represents pansusceptible *Salmonella* and the red piece represents resistance to 3 or more classes of antimicrobials (MDR). Total number of the *Salmonella* positive samples and total faecal samples tested are shown below each pie in the text box. Percentages of pansusceptible and MDR isolates are shown inside the circle.

In total, *Salmonella* was recovered from 566 out of 1,040 faecal samples. The mean *Salmonella* prevalence before the antimicrobial treatments began (day 0) was 75.0% (95% CI: 67.9–81.2%), with 132 out of 176 samples testing positive. Among the two groups of all cattle treated with CCFA (All-CCFA/no CTC, All-CCFA & CTC), the prevalence of *Salmonella* was 34.1% (15 positive out of 44 samples) and 27.3% (12 positive out of 44 samples) on day 4, respectively. By day 14, the *Salmonella* prevalence in cattle receiving no subsequent CTC treatment (All-CCFA/no CTC; 31 positives out of 43 samples, 72.1%) was similar to that of those steers with least antimicrobial exposures (1-CCFA/no CTC; 35 positives out of 44 samples, 79.5%) as shown in Fig. 2 (second row). When CTC treatment followed CCFA treatment, the prevalence dropped even further to 16.3% by day 14 (7 out of 43 positive). By day 26, the prevalence in both CTC treatment groups (1-CCFA & CTC, All-CCFA & CTC) returned to 47.1% (40 out of 85 positive). By comparison, the overall mean prevalence in steers in those pens that received the least antimicrobial treatment (1-CCFA & no CTC) was estimated at 72.7% (95% CI: 66.8–78.0%) throughout the study period.

### Descriptive statistics on *Salmonella* isolates resistant to antimicrobials

The total number of *Salmonella* isolates and the percentage of MDR *Salmonella* (defined as resistant to ≥3 antimicrobial classes) for each sampling day by antimicrobial treatment groups are shown in an inner circle of the Fig. 2. Among the steers with the least exposure of antimicrobial treatment, the vast majority of isolates remained pansusceptible throughout the study period, although 3.2%, 5.7%, and 3.8% multidrug resistant *Salmonella* isolates were detected on days 4, 14 and 20, respectively (Fig. 2, first row). Among the group that all cattle treated with CCFA on day 0, 26.7% of *Salmonella* isolates were MDR on day 4 and 22.7% were MDR on day 8; however, MDR prevalence thereafter declined by day 14 (Fig. 2, second row). In the treatment group treated with CTC (1-CCFA & CTC) starting from day 4, pansusceptible isolates decreased and MDR isolates increased (73.3%) on day 8 and further increased to 80% and 100% on days 14 and 20, respectively (Fig. 2, third row). By the end of the study (day 26), 50% of the isolates in this latter group remained as MDR. Among animals sequentially receiving both CCFA and CTC treatment, the pattern of MDR dynamics resembled that of the treatment groups receiving CTC (Fig. 2, fourth row).

### Mixed effects logistic regression model of *Salmonella* prevalence and MDR *Salmonella* prevalence

**Figure 3:**
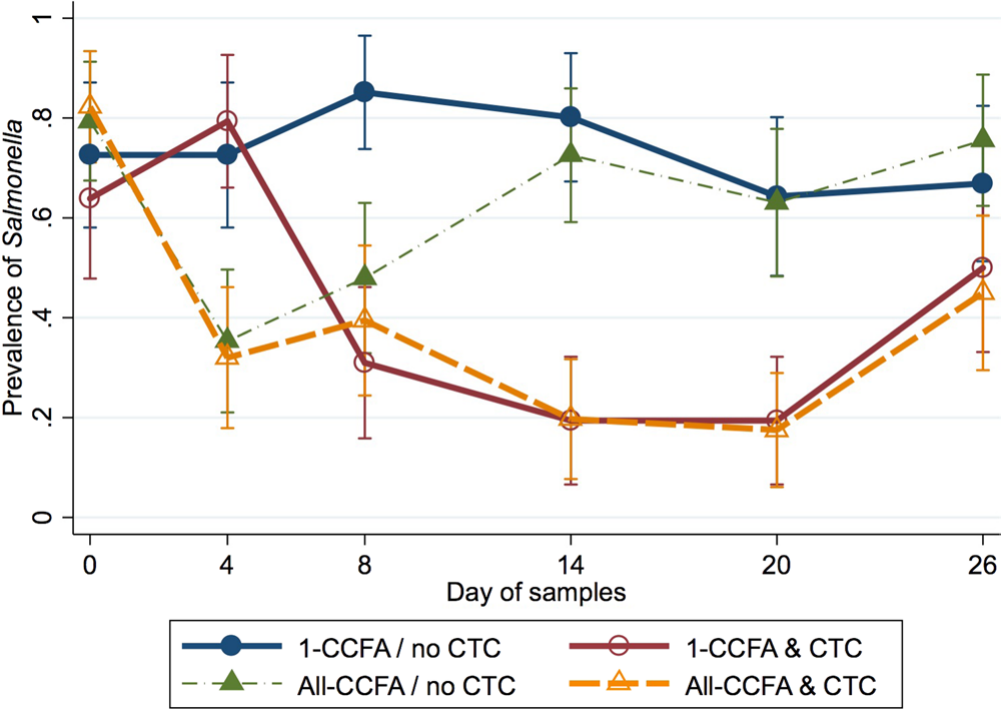
Modelled marginal mean prevalence of *Salmonella* by day and treatment. Modelled marginal mean prevalence of *Salmonella* and 95% confidence intervals in cattle faecal samples by sample day and treatment group. Solid lines represent treatment groups in which a single CCFA treated steer was mixed within an otherwise untreated group while dashed lines represent groups in which all steers received CCFA treatment.

CCFA and CTC treatments were coded as binary variables, with 0 (no treatment) used as the referent category. Days were 0, 4, 8, 14, 20, and 26, with day 0 used as the referent. CCFA treatment effects were highly significant on days 4 (p < 0.004) and 8 (p < 0.003). CTC treatment significantly decreased the prevalence of *Salmonella* on days 8 (p < 0.004), 14 (p < 0.001), and 20 (p < 0.024) when compared to day 0 (Fig. 3).

**Figure 4:**
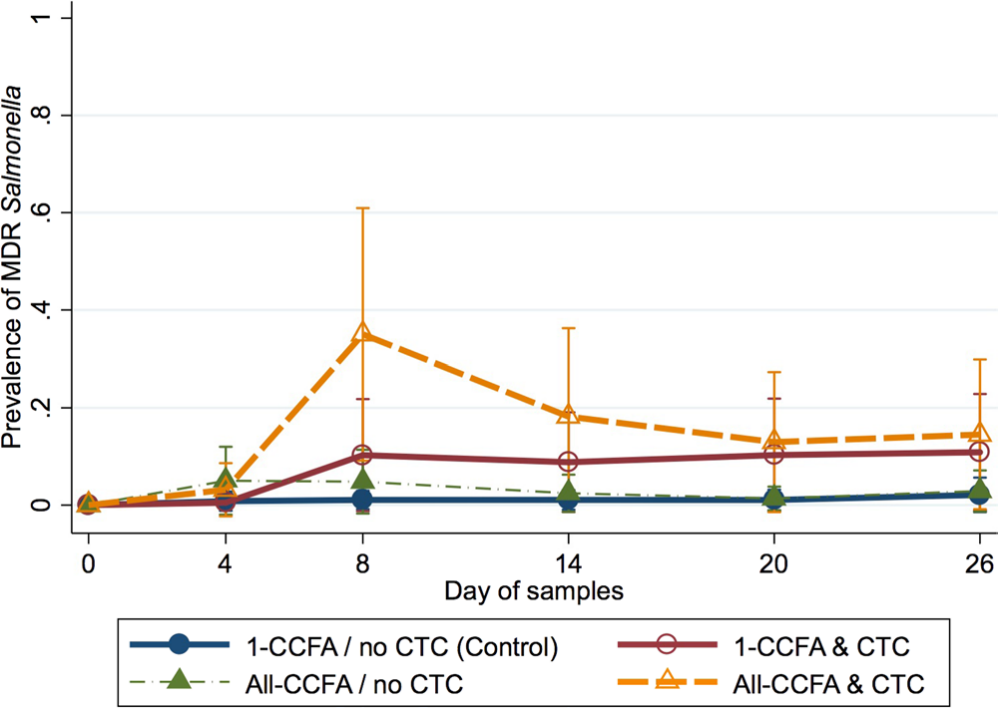
Modelled marginal mean prevalence of MDR *Salmonella* by day and treatment. Modelled marginal mean prevalence of *Salmonella* and 95% confidence intervals in cattle faecal samples by sample day and treatment group. Solid lines represent treatment groups in which a single CCFA treated steer was mixed within an otherwise untreated group while dashed lines represent groups in which all steers received CCFA treatment.

The prevalence of MDR *Salmonella* was modelled similar to that of overall *Salmonella* prevalence, as above, except that day 4 was used as a referent category. This is because MDR isolates first appeared on day 4, yielding unstable model parameters for day 0. Interaction between CTC treatment and day was responsible for an increase of MDR *Salmonella* from day 8 (p < 0.008) to day 26 (p < 0.041) (Fig. 4). CCFA treatment on day 0 further increased MDR *Salmonella* probability on day 8 in the CCFA/CTC treated groups, compared with the group treated only with CTC. A slight increase of MDR *Salmonella* was seen on day 4 in the CCFA-only treated group, compared to the referent group (1-CCFA & no CTC); however, this was not significant (P > 0.05).

### Identification of serotypes and MLST

**Figure 5:**
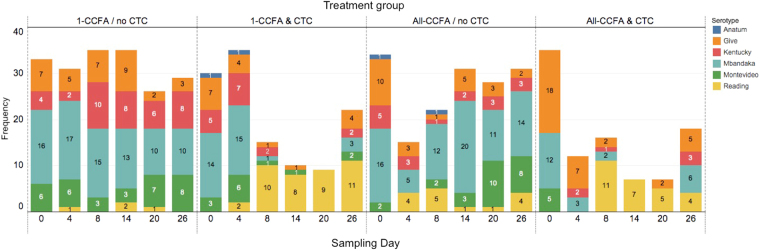
Number of *Salmonella enterica* serotypes by treatment group and sample day. Six serotypes (Anatum, Give, Kentucky, Mbandaka, Montevideo, and Reading) were found among tested faecal samples. Numbers shown in the bars are the number of isolates for each serotype by treatment group across both trial replicates.

*Salmonella* serotypes were identified by whole-genome sequencing using the SeqSero pipeline^34^, which determines the serotype based on the sequence of the O-antigen gene cluster and H1 and H2 antigens. Six serotypes were detected; the most common serotype was *Salmonella* Mbandaka (38.0%), followed by *S*. Give (19.1%), *S*. Kentucky (13.6%), *S*. Reading (15.2%), *S*. Montevideo (13.4%), and *S*. Anatum (0.7%) as shown in Table 1. Serotypes and MLST were matching completely and no diverse serotypes were detected from any single MLST (Table 1). *Salmonella* Reading was detected starting on day 4, and increased greatly by day 8 in the All-CCFA/no-CTC groups, and especially in the 1-CCFA & CTC and All-CCFA & CTC treatment groups (Fig. 5) where it extended well past day 8. Five serotypes, except *S*. Reading, were identified on day 0. In the 1-CCFA/no CTC group, the composition of serotypes remained similar throughout the study. The *S*. Reading isolate detected on day 4 in the 1-CCFA/no CTC group was derived from the single steer that received CCFA and was mixed in the group comprised of 10 other non-CCFA-treated cattle; however, the isolates from day 14 and 20 were from the steers that were not treated with antimicrobials. In the All-CCFA/no CTC group, susceptible serotypes decreased on day 4 while *S*. Reading was increasingly present. In the 1-CCFA & CTC group, all isolates were *S*. Reading on day 20; however, susceptible serotypes had recovered by day 26. The All-CCFA & CTC group exhibited less serotype diversity. *S*. Montevideo was detected only on day 0 in this group. More *S*. Reading were isolated in the treatment groups that received CTC. Serotype distribution was similar between replicates 1 and 2, except that *S*. Anatum was identified only in replicate 1 (Fig. S1). *S*. Mbandaka were in the majority and *S*. Reading was the least detected serotype in the 1-CCFA & no CTC group (Table 2). *S*. Give were in the majority followed by *S*. Reading in All-CCFA & CTC group. In 1-CCFA & CTC group, 33.1% were *S*. Reading.

**Table 1:** **Multi-Locus Sequence Type (MLST) and serotypes of the** ***Salmonella*** **isolates**.

Serotype	Number of isolates	Antigenic profile from Seqsero				MLST	Gene Allele						
		O	H1 (*fli*C)	H2 (*flj*B)	Predicted profile		*aro*C	*dna*N	*hem*D	*his*D	*pur*E	*suc*A	*thr*A
Anatum	4 (0.7%)	O-3,10	e,h	1,6	3,10:e,h:1,6	64	10	14	15	31	25	20	33
Kentucky	77 (13.6%)	O-8	i	z6	8:i:z6	198	76	14	3	77	64	64	67
Mbandaka	215 (38.0%)	O-7	z10	e,n,z15	7:z10:e,n,z15	413	15	70	93	78	113	6	68
Montevideo	76 (13.4%)	O-7	g,m,s	—	7:g,m,s:-	138	11	41	55	42	34	58	4
Give	108 (19.1%)	O-3,10	l,v	1,7	3,10:l,v:1,7	654	111	47	49	42	12	58	3
Reading	86 (15.2%)	O-4	e,h	1,5	4:e,h:1,5	1628	46	60	10	9	6	12	17

**Table 2:** Proportion of *Salmonella* serotype and AMR phenotype by treatment group.

Serotype	**Serotype per Treatment Group**				**AMR Phenotype**
	**1-CCFA/no CTC**	**1-CCFA & CTC**	**All-CCFA/no CTC**	**All-CCFA & CTC**	
Anatum (n = 4)	0.0% (0)	1.7% (2)	1.2% (2)	0.0% (0)	Pansusceptible: 100.0% (4)
Give (n = 108)	17.5% (33)	14.0% (17)	14.9% (24)	35.8% (34)	Pansusceptible: 100.0% (108)
Kentucky (n = 77)	20.1% (38)	13.2% (16)	10.6% (17)	6.3% (6)	Pansusceptible: 98.7% (76), **STR-SUL-TET: 1.3% (1)**
Mbandaka (n = 215)	42.9% (81)	27.3% (33)	48.4% (78)	24.2% (23)	Pansusceptible: 100.0% (215)
Montevideo (n = 76)	17.5% (33)	10.7% (13)	15.5% (25)	5.3% (5)	Pansusceptible: 100.0% (76)
Reading (n = 86)	2.1% (4)	33.1% (40)	9.3% (15)	28.4% (27)	**AMP-AUG2-AXO-FOX-TIO-SOX-STR-CHL-TET: 96.5% (83), AMP-AUG2-AXO-FOX-TIO-STR-CHL-TET: 3.5% (3)**
Total (n = 566)	100.0% (189)	100.0% (121)	100.0% (161)	100.0% (95)	

For quality control purposes, 2 isolates randomly chosen from each of the 5 serotypes identified by whole-genome sequencing (WGS) were sent to the National Veterinary Services Laboratories for traditional serotyping. All traditional serotyping results matched exactly with sequence based serotyping.

### Associations of phenotypic antimicrobial resistance profile and serotypes of *Salmonella*

All isolates (n = 566) were tested against a standard NARMS panel including 14 antimicrobials arising from 9 classes. Serotype and resistant phenotype were significantly associated (p < 0.05). All of the *S*. Anatum, *S*. Give, *S*. Mbandaka and *S*. Montevideo were phenotypically pansusceptible. Nearly all (96.5%) of the *S*. Reading isolates had at least the penta-resistant profile, ACSSuT (resistant to ampicillin, chloramphenicol, streptomycin, sulphonamides, and tetracycline), with additional resistance to 3^rd^ generation cephalosporins ceftiofur and ceftriaxone (Table 2). The other 3.5% isolates detected from 1-CCFA & CTC group were not resistant to sulphonamides. The resistance profiles were AMP-AUG2-AXO-FOX-TIO-STR-CHL-TET for 8 antimicrobials and AMP-AUG2-AXO-FOX-TIO-FIS-STR-CHL-TET for 9 antimicrobials. One *S*. Kentucky isolate from 1-CCFA & CTC group was resistant to 3 antimicrobials: STR, FIS, and TET (Table 2). The proportion of *S*. Reading was significantly higher (p < 0.05) in the CTC treatment group than in the groups without CTC treatment. No resistance to azithromycin, ciprofloxacin, gentamicin, or nalidixic acid was detected.

### Serotype pattern per individual animal

**Figure 6:**
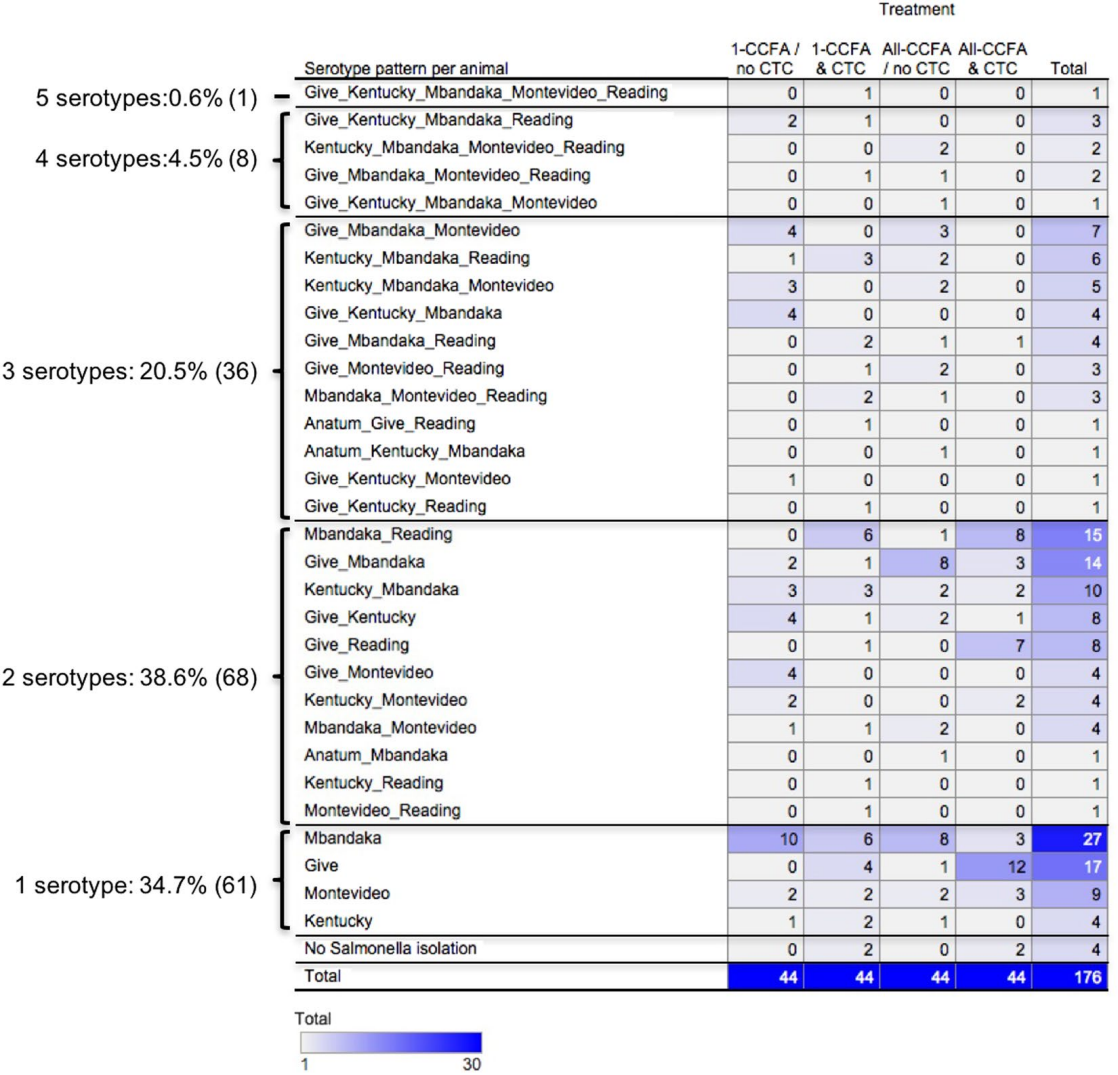
Total number of animals harboring each serotype pattern across all sampling days. Serotype patterns were clustered per treatment group across from different sampling days. Darker blue indicates more animals with specific serotype patterns; conversely, lighter blue shaded serotype combinations had fewer animals.

We aggregated the *Salmonella* serotypes that were detected in the same animal from different sampling days to identify the serotype patterns (Fig. 6). In one steer, 5 serotypes (*S*. Give, Kentucky, Mbandaka, Montevideo, and Reading) were detected across the different days. In 4.5% (8 steers), 4 serotypes with different serotype patterns were detected. Three serotypes were detected from 20.5% (36 steers) and 2 serotypes were detected from 38.6% (68 steers). A single serotype was detected from 34.7% (61 steers). No steers were detected with *S*. Reading alone; that is, it was always detected with other serotypes across different days. Four steers were not detected as harbouring *Salmonella* throughout the study period.

## Discussion

This randomized controlled longitudinal field trial has clearly demonstrated that the use of antimicrobials shifts the antimicrobial resistance status of the *Salmonella* population by selecting for MDR *Salmonella* and against the pansusceptible *Salmonella* serotypes that are highly prevalent in beef cattle in this region of the USA. Since resistance phenotype and *Salmonella* serotype are so strongly associated, this effectively means that antimicrobial use selects for serotype. Chlortetracycline treatment alone, or CTC with prior treatment with CCFA, decreased the overall prevalence of *Salmonella*; further, each of these treatments increased the proportion of MDR *Salmonella* that remained. Ceftiofur treatment by itself did reduce the prevalence of *Salmonella*; however, such a reduction was relatively transient. The sole MDR phenotypic pattern was virtually identical among the *S*. Reading (Table 2). Six *Salmonella* serotypes, which have previously been reported in feedlot cattle and the feedlot environment, were detected in the faecal flora of these cattle^37–40^. As a point of reference, *Salmonella* Anatum, Montevideo, and Kentucky accounted for 50.4% of the serotypes detected in the National Animal Health Monitoring Systems (NAHMS) Feedlot 2011 study^39^.

We found a high prevalence of *Salmonella* in the faeces of feedlot cattle prior to antimicrobial treatment. Previous attempts to determine the dynamics of *Salmonella* in experimental studies were likely not successful due to a low prevalence of overall and resistant *Salmonella* in the study population; thus requiring a vast number of animals on trial to have enough power for the analyses^22,25^. Even in large-scale observational studies the prevalence can vary a great deal, illustrating that in many U.S. locations the prevalence is very low. The NAHMS Feedlot 2011 study had earlier shown that the overall pen-level prevalence of *Salmonella* in feedlot cattle across the United States was 35.6%, with 9.1% sample (cattle)-level prevalence^39^. Our study successfully showed that *Salmonella* population changes in response to antimicrobial treatments, in part due to the high prevalence at the beginning of the study (i.e., 70 + %). Some of the factors explaining the high initial prevalence in our study include geography (southern United States) and season (August-October). Several studies have shown a seasonal variation in *Salmonella* prevalence, such that sampling during the summer months yields the highest percentage of *Salmonella* positive samples^41–44^. One study from North Dakota found 62.2% prevalence of *Salmonella* in the tested herd during spring months, but only one serotype *S*. Typhimurium var. Copenhagen was isolated from the entire herd^45^. It is possible that if our study was conducted during colder weather, such an obvious change in the *Salmonella* population might not be observed. Regardless, the population dynamics of *Salmonella* associated with the antimicrobial treatments may be similar. The *Salmonella* prevalence and serotype populations differ by region, country, and ambient environment, which imply that the resistance phenotype will vary at the same time. Therefore, our results may not be necessarily be generalized to feedlots on other continents, as one example.

One-time CCFA treatment (as is typically used to control respiratory disease in beef feeder cattle) on day 0 reduced the overall prevalence of *Salmonella* in faeces. CCFA is not labelled for the control of *Salmonella* in cattle; however, its broad-spectrum nature, presence in multiple tissues, and effectiveness against Gram-negative bacteria appears to result in a temporal decrease of *Salmonella* prevalence immediately following treatment. The dose of CCFA used in the current study was for the treatment and control of bovine respiratory diseases such that the serum concentration is maintained over the minimum inhibitory concentration (0.2μg/ml) for up 10 days following a single-dose administration. A previous study illustrated that an extra-label regimen of ceftiofur decreased the detection and quantity of *Salmonella* and effectively treated salmonellosis in neonatal calves^46^. In our study, CCFA treatment also increased the proportion of MDR *Salmonella* on days 4 through 8. Despite only receiving a single dose of CCFA, by the end of the study (day 26) 12.5% of isolated *Salmonella* remained multidrug resistant (Fig. 2). Few observational or experimental studies have explored the association between antimicrobial use (including ceftiofur) and the temporal dynamics of resistant *E*. *coli* and *Salmonella* in cattle and pigs^22,24,26,28,30,47–50^. With the follow-up of CTC treatment to earlier CCFA treatment, MDR isolates increased further to 75% of total *Salmonella* by day 14. We chose to explore day 14 in order to examine the status of antimicrobial resistant *Salmonella* in cattle faeces on the first day post-treatment that the animals were eligible to be sent to a slaughterhouse based on residue avoidance; that is, because the labelled slaughter withholding time of CCFA is 13 days. Our findings indicate that at the point at which this compliance requirement is met, MDR *Salmonella* prevalence persists far above the baseline starting values, a finding that has not been previously reported. However, we are well aware that such a scenario is highly unlikely in real feedlot settings; that is, CCFA treatment at the whole pen level is extremely unlikely to occur anytime close to when cattle are sent to slaughterhouse. Typically, fed cattle would instead be sent to slaughter at least 6 months post-arrival. To understand longer-term dynamics, additional studies would be needed to further investigate the effects of CCFA and CTC treatments over periods of many months.

While CTC has been used for several decades in animal agriculture for prevention and growth promotion purposes, the effects of therapeutic doses of CTC on the prevalence and resistance of *Salmonella* in cattle have not been studied extensively. CTC was added to the feed (as a top-dress) for 3 pulses of 5 continuous days, with a one-day interval in between. This regimen was designed to observe the maximum effect of CTC, to follow the product label in the U.S., and to be consistent with a previous study published by Platt *et al*.^30^. CTC treatment alone reduced *Salmonella* prevalence to the same levels as cattle first injected with CCFA and then subsequently treated with CTC. This further suggests that initial CCFA treatment did not have a significant long-term effect on *Salmonella* prevalence. However, it remains possible that the initial CCFA may have selected for MDR *Salmonella*, which will be discussed later. The effects of CTC given at therapeutic or subtherapeutic doses on pathogens including *Salmonella* have been reported in pigs^51,52^. Although not statistically significant, pulsed CTC feeding during the finishing period lowered *Salmonella* prevalence in pigs^51^. Wells *et al*. have shown that CTC supplemented in the diet reduced the prevalence of both *Campylobacter* and Shiga-toxin producing *E*. *coli* ^52^. Prevalence of *Salmonella* was at a negligible level in their study. A study by Agga *et al*. found that one-set of 5-day CTC in-feed treatments transiently increased tetracycline resistant *E*. *coli* concentration in the faecal swabs of feedlot cattle. Importantly, generic *E*. *coli* concentration in the CTC treatment group remained the same, thus indicating that tetracycline resistant *E*. *coli* effectively replaced the susceptible *E*. *coli* population^31^.

Previous work by our group illustrated an inexplicable paradoxical reduction of ceftiofur resistant *E*. *coli* via CTC treatment (despite mechanistic potential for co-location via *tet*(A) and *bla*_cmy-2_ genes housed on a single IncA/C plasmid in many cattle *E*. *coli*). We explored the potential that this might serve as a potential intervention to reduce ceftiofur resistance by instead favouring singly resistant *E*. *coli* strains harbouring the *tet*(B) gene on the bacterial chromosome^27,30^. In our current study, three pulsed (intermittent) CTC treatments displayed a stronger selection pressure on MDR *Salmonella* than CCFA treatment alone, which was completely the opposite of the previous published work exploring the effects of these same regimens in *E*. *coli* populations^27^. In that study, faecal *E*. *coli* from CCFA administrated pens showed more resistance to a full range of antimicrobials than in cattle from the pens administered only CTC (the latter effect was negligible)^27^. As mentioned, while Agga *et al*. showed that a 5 day single-pulsed CTC treatment increased tetracycline resistant *E*. *coli* in faecal swabs of beef cattle at 5 days-post-treatment, no differences in cephalosporin resistant *E*. *coli* were found^31^. It is possible that the resistant *E*. *coli* population were different between these two studies. Comparatively phenotypic resistance patterns and sequence type (or PFGE patterns) of *E*. *coli* in earlier studies of food animals are often diverse, while *Salmonella* are often either pansusceptible or else MDR (assuming they are present at all)^26,27,38,53^. Importantly, in *Salmonella*, drug resistance phenotypes and serotypes are much more highly associated than is seen across the more prevalent and diverse populations of *E*. *coli* ^38,54,55^. These differences in resistance patterns likely indicate that *Salmonella* and *E*. *coli* are not readily sharing their resistance elements in the cattle gut microbiome^53^; conversely, they also might not be sharing the same ecological niches in the varying cross-sections of intestinal regions^56^. One previous experimental study suggested that the use of ceftiofur in dairy cattle did not promote the transfer of *bla*_CMY-2_ coding plasmids among *Salmonella* and commensal *E*. *coli* in calves inoculated with plasmid-bearing bacteria and in dairy herds using ceftiofur^24^. However, another study found that the presence of an inflammatory condition in the gut could boost horizontal gene transfer between *Salmonella* and *E*. *coli* ^57^, something that is often lacking in studies involving healthy animals. Although limited to phenotypic resistance, our study reveals that *Salmonella* and *E*. *coli* isolated from the same gut microbiome did not share similar resistant patterns, either at baseline or during specified treatment periods and across treatment regimens^27^.

We detected 6 serotypes in our study population; in decreasing prevalence, these were *Salmonella* Mbandaka, Give, Kentucky, Reading, Montevideo, and Anatum. The serotypes detected in this study were consistent with those published in previous studies and isolated from the lymph nodes, hides, and faeces of feedlot and dairy cattle from the same region of Texas^38,43,58–61^. *Salmonella* serotypes often appear to adapt to specific animal hosts. Among human clinical isolates reported to the CDC, *S*. Montevideo was 10th among frequently reported serotypes in 2013^62^. In the current study, *S*. Mbandaka (serogroup C1) was the dominant serotype followed by Give (serogroup E1). The dominance of certain serotypes has been observed in other studies in feedlot cattle as well^60,61^. However, it is possible that *Salmonella* enrichment via RV media may bias the detection towards serogroups C1, C2, and E^63^. We detected *S*. Reading, which belongs to serogroup B, but none of these were pansusceptible. It remains a possibility that pansusceptible *S*. Reading isolates were not detected in our study due to enrichment bias or else the resistant *S*. Reading population was stable in the gut microbiota but were not able to outcompete the susceptibles prior to antimicrobial pressure. Our study has illustrated that serotype and MDR may be strongly associated. Most (96.5%) *S*. Reading isolates had the ACSSuT resistance profile, while the other serotypes did not; this agrees with previous work that suggests that *S*. Reading is more likely to be of the ACSSuT resistance phenotype than other serotypes^43,59^. It remains unclear why certain serotypes are more highly resistant to antimicrobials, while others are not. However, all of these cited studies suggest that the antimicrobial susceptibility of the various *Salmonella* serotypes might have been determined not only by their genetic predispositions, but also by coexisting serotypes as well as environmental factors, such as antimicrobial selection pressure.

The first and second replicates of these cattle trials were conducted at the same feedlot with a one-month interval between the studies. Cattle were housed in porous-fenced pens through which contact could be made with cattle in adjacent pens. Treatments were randomly assigned to balance the contact potential across pens; however, sterility could not be maintained between pens in such a field setting as is standard in the beef feeding industry. The serotype distribution of the first and second replicates was similar throughout the study and no *S*. Reading was found on day 0, even in the second replicate (Supplementary Fig. S1). This latter point strongly suggests that MDR *Salmonella* transiently detected solely due to the antimicrobial pressure applied during this trial. All the steers that were detected with *S*. Reading on at least one sampling day also had other serotypes isolated during at least one other sampling day (Fig. 6). Since these serotypes were not detected at the same time point (i.e., since we limited the number of colonies we assayed from each plate), it does not necessarily mean that the steers were infected with 2 or more serotypes at once; however, this point does support the idea that *S*. Reading likely co-resided with other serotypes under “normal” non-antimicrobial selection pressure conditions, and was more readily detected after the antimicrobial treatments since other pansusceptible *Salmonella* serotypes were selected against by antimicrobials.

The steer isolated with 5 different serotypes on 5 different days illustrates the complexity of *Salmonella* colonization in cattle; this is consistent with another study in dairy cattle that showed multiple serotype colonizations per animal^38,64,65^. In agreement with our study, confirmation of *Salmonella* infection or colonization sometimes requires multiple testing methods, enrichment, and sequential sampling time-points. Four steers in our study were negative for *Salmonella* throughout the whole study period (26-days); even after this, these can be interpreted still as being of rare or intermittent shedding of *Salmonella* or as being truly negative for *Salmonella* colonization.

One limitation of this study is that we terminated the study on day 26 and thus did not track the cattle to the age at which they would be sent to the slaughterhouse, and whose faecal, hide, and lymph node *Salmonella* populations would represent a greater potential threat to public health. The feedlot is the final production stage for beef cattle, and thus represents a more proximate source of MDR *Salmonella* contamination at the slaughterhouse than occurs earlier in the beef production cycle^66^. In the future, we plan to conduct a similarly designed randomized controlled study with follow-up extending to the slaughterhouse; that is, approximately 150 days post-treatment. We currently hypothesize that any temporal antimicrobial treatment effects will wane over such an extended period of time, yielding few, if any, differences among the treatment groups by the time animals are ready for slaughter as shown in previous studies in *E*. *coli* population^26,31^.

In conclusion, we found that CTC and CCFA treatments dramatically decreased overall *Salmonella* prevalence in feedlot cattle; however, multi-drug resistant *Salmonella* strains were selected (or, detected) instead of those that comprised the original antimicrobial-susceptible *Salmonella* population. *Salmonella* serotypes and antimicrobial resistance phenotypes displayed strong associations and suggest that specific serotypes may be more likely to carry MDR genes. Although it was transient, this study indicates that the use of CCFA and CTC exerts a strong selection pressure on the *Salmonella* populations in the gut of feedlot cattle; therefore, judicious use of antimicrobials is necessary for beef cattle feeding operations to aid in preventing increased levels of MDR *Salmonella* being present in cattle destined for slaughter.

## Electronic supplementary material

Supplementary information
