# Supplementary material for: Population dynamics of enteric Salmonella in response to antimicrobial use in beef feedlot cattle
Source: Sci Rep. 2017 Oct 30;7:14310. doi: 10.1038/s41598-017-14751-9 (PMC5662634; doi:10.1038/s41598-017-14751-9)
Supplement: Supplementary file 1 — Supplementary information [file 41598_2017_14751_MOESM1_ESM.doc]

**Supplementary Information**

Population dynamics of enteric *Salmonella* in response to antimicrobial use in beef feedlot cattle

Naomi Ohta, Keri N. Norman, Bo Norby, Sara D. Lawhon, Javier Vinasco, Henk den Bakker, Guy H. Loneragan, H. Morgan Scott


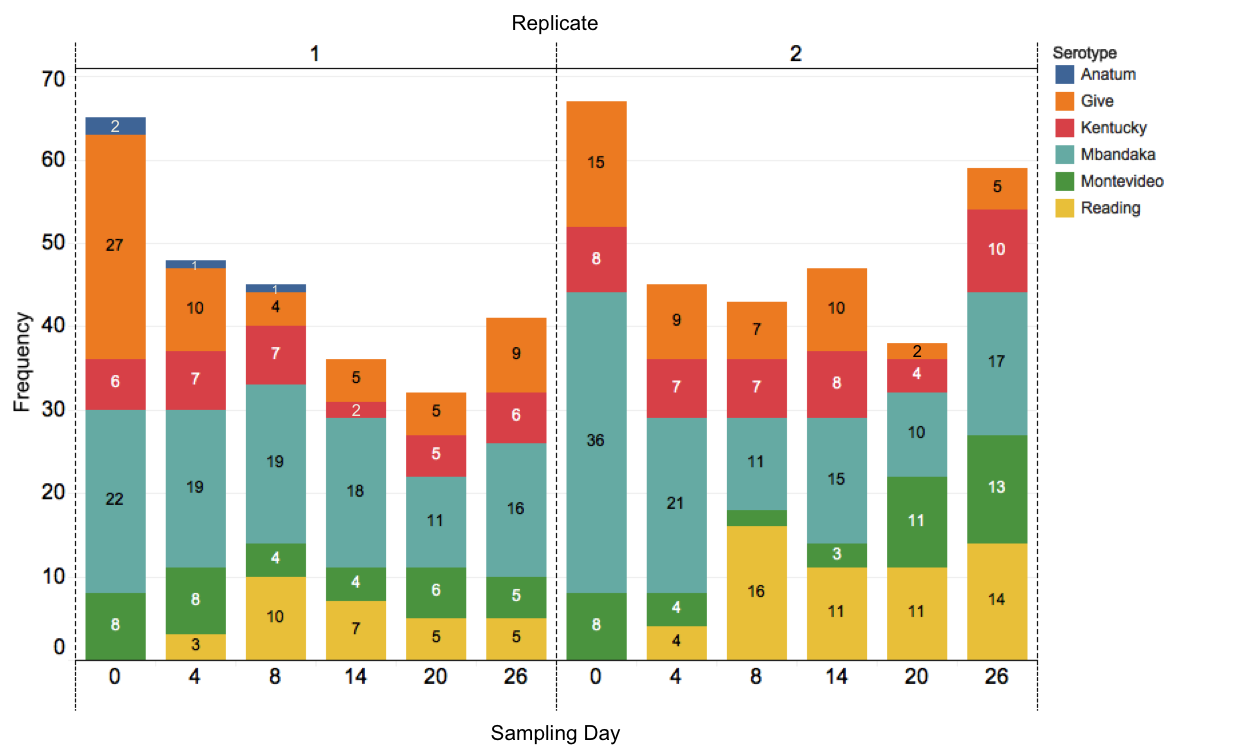


**Supplementary Figure S1. Distribution of *Salmonella enterica* serotypes by replicate and day of sampling across all treatment groups.**
